# miR-877-3p targets Smad7 and is associated with myofibroblast differentiation and bleomycin-induced lung fibrosis

**DOI:** 10.1038/srep30122

**Published:** 2016-07-22

**Authors:** Cong Wang, Shen Gu, Honghui Cao, Zutong Li, Zou Xiang, Kebin Hu, Xiaodong Han

**Affiliations:** 1 Immunology and Reproduction Biology Laboratory & State Key Laboratory of Analytical Chemistry for Life Science, Medical School, Nanjing University, Nanjing, Jiangsu 210093, China; 2 Jiangsu Key Laboratory of Molecular Medicine, Nanjing University, Nanjing, Jiangsu 210093, China; 3 Department of Microbiology and Immunology, Mucosal Immunobiology and Vaccine Research Center, Institute of Biomedicine, University of Gothenburg, Gothenburg, Sweden; 4 Department of Medicine, Division of Nephrology, Penn State University College of Medicine, Hershey, Pennsylvania 17033, USA.

## Abstract

Myofibroblast differentiation of lung resident mesenchymal stem cells (LR-MSC) plays an important role in idiopathic pulmonary fibrosis. By comparing the expression profiles of miRNAs before and after myofibroblast differentiation of LR-MSC, we identified miR-877-3p as a fibrosis-related miRNA. We found that miR-877-3p sequestration inhibited the myofibroblast differentiation of LR-MSC and attenuates bleomycin-induced lung fibrosis by targeting Smad7. Smad7, as an inhibitory smad in the TGF-β1 signaling pathway, was decreased in the myofibroblast differentiation of LR-MSC and up-regulation of Smad7 could inhibit the differentiation process. Our data implicates a potential application of miR-877-3p as a fibrosis suppressor for pulmonary fibrosis therapy and also as a fibrosis marker for predicting prognosis.

Idiopathic pulmonary fibrosis^1^ is a chronic lung disease characterized by excessive myofibroblast proliferation and extracellular matrix (ECM) deposition that disrupts the normal architecture of the pulmonary alveoli. IPF patients have a median survival of 2.5–3.5 years after diagnosis^2^. Despite extensive investigation, the pathogenesis of IPF is not fully understood and no effective drug treatment is available at present. It is thus important to unravel the mechanisms underlying the pathogenesis of pulmonary fibrosis, which may facilitate the development of effective treatment strategies.

Accumulating evidence suggests that lung remodeling by mesenchymal-derived cells, mainly myofibroblasts, may subsequently result in destruction of the lung architecture^3^. Myofibroblasts are specialized fibroblasts that possess the morphologic and biochemical characteristics of both fibroblasts and smooth muscle cells, which have been proposed as the main source of ECM within the impaired lung of patients with IPF^4^. Therefore, defining the origins of these proliferative myofibroblasts and suppression of their accumulation during pulmonary disease remains an effective therapeutic strategy.

More and more studies have also demonstrated that mesenchymal stem cells (MSCs) are crucial for the development of pulmonary fibrosis. MSCs are precursors of myofibroblasts and can be induced to differentiate into many other cell types. The most well-researched MSCs are bone marrow MSCs (BM-MSCs), which are the easiest to obtain and have a great potential for treating various diseases^5^. However, it is generally believed that tissue MSCs exist in almost all tissues including the lungs^6^, and an increasing number of studies have shown that lung resident mesenchymal stem cells (LR-MSCs) may be more efficient than BM-MSCs from a therapeutic perspective^7^. These LR-MSCs regulate the repair process by differentiation into several cell types, which may participate in lung repair or contribute to the development of pulmonary diseases.

More importantly, the behavior of LR-MSCs is highly sensitive to the microenvironment to which these cells are exposed^8^. Transforming growth factor β1 (TGF-β1) is highly expressed in pulmonary fibrosis and generally acknowledged as the master regulator of myofibroblast differentiation. It has recently been shown that TGF-β1 expression within the lungs of premature infants stimulates LR-MSCs to differentiate into myofibroblasts^9^. Ligand binding to TGF-β1 receptor causes phosphorylation of cytoplasmic Smad2 or Smad3, promoting Smad hetero complex formation and translocation to the nucleus to directly regulate the transcription of target genes^10^. It is interesting that Smad7 is an inhibitory Smad that blocks the function of Smad2 and Smad3, consequently inhibiting the binding to the activated receptors and exerting its negative effect on TGF-β1/Smad signaling^11^. More and more recent studies have indicated that LR-MSCs could be triggered by TGF-β1 to differentiate into myofibroblasts, contributing to disease progression^12^. However, the mechanism of myofibroblast differentiation of LR-MSCs remains unclear.

MicroRNAs (miRNAs) are a class of noncoding RNAs consisting of processed products of approximately 22-25 nucleotides in length that play important roles in regulating posttranscriptional gene silencing via base pair binding to the sequences on the 3′-untranslated regions (UTRs) of mRNA^13^. miRNA has crucial functions in diverse cellular processes, including cell differentiation and proliferation, and tissue development and repair^14^. Differential expression of miRNAs between IPF lungs and control has been revealed. Recent studies have demonstrated that miRNAs play important roles in lung fibrosis which represents a new layer of gene expression regulation at the post-transcriptional level^15^. Despite a clear, important role of LR-MSCs in pulmonary fibrosis, the involvement of miRNAs and the roles of miRNAs in TGF-β1-induced myofibroblast differentiation of LR-MSCs are uncertain.

In a previous study, we have identified Sca-1^+^ CD45^−^CD31^−^ cells isolated from lung tissues as LR-MSCs^6^. In this study, we investigated the role of miRNA in TGF-β1-induced myofibroblast differentiation of LR-MSCs and tried to determine whether this could provide a mechanistic explanation for the pathogenesis of lung fibrosis, which may facilitate the development of effective treatment strategies.

To our knowledge, this is the first study that demonstrates altered miRNA expression in LR-MSCs after myofibroblast differentiation. We also investigated the regulation of specific miRNAs in TGF-β signaling pathways and pulmonary fibrosis development in a mouse model of pulmonary fibrosis.

## Results

### miRNAs are differentially expressed in LR-MSCs

**Figure 1:**
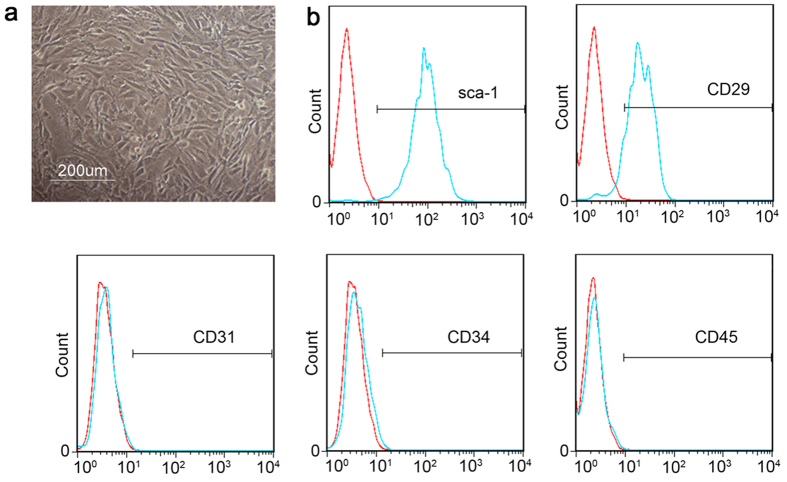
The phenotypes of the lung resident mesenchymal stem cells (LR-MSCs) were confirmed by microscopy and flow cytometry. Mouse LR-MSCs were collected and cultured for 7 days. (**a**) Cell morphology was examined by a standard light microscope. (**b**) Representative flow cytometric analysis of the cell surface markers of LR-MSCs is shown. The negative control (red) and indicated surface marker (blue) was merged, 1 × 10^4^ cells were counted per experiment.

**Figure 2:**
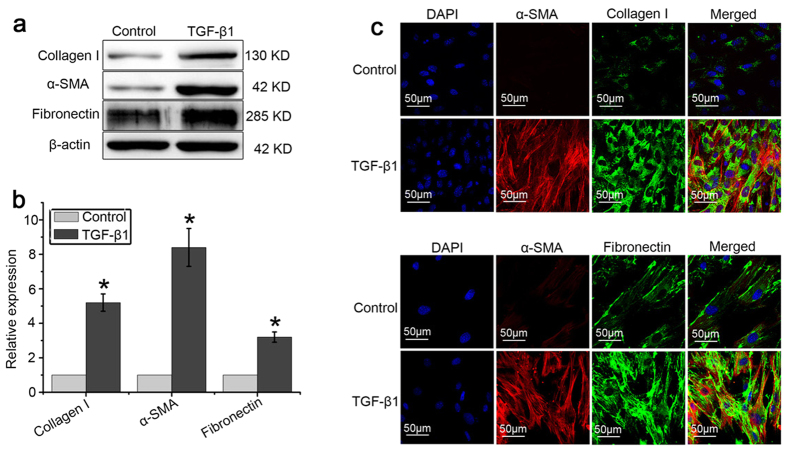
TGF-β1 induced LR-MSCs to differentiate into myofibroblasts. LR-MSCs were cultured with or without TGF-β1 (10 ng/ml) for 7 days followed by measurement of myofibroblast markers on LR-MSCs. (**a**,**b**): Expression of collagen I, α-SMA, and fibronectin on LR-MSCs was examined by western blotting. Representative gel electrophoresis bands are shown (**a**), and protein expression levels were quantified by densitometry and normalized to the expression of β-actin (**b**). Densitometry data are shown as mean ± SD. Statistical analysis was performed using one sample Student t-test. *P < 0.05 vs. control. (**c**) Expression of collagen I, α-SMA and fibronectin in LR-MSCs was measured by immunofluorescent microscopy (×600).

Following culture for 7 days, the cells isolated from mouse lungs demonstrated the morphology of a long spindle, spiral, and radial arrangement (Fig. 1a). These cells expressed SCA-1 and CD29, but not CD31, CD34 or CD45 (Fig. 1b). Taken together, these results suggest that the cells possessed the main features of mesenchymal stem cells. Following treatment with TGF-β1 for 7 days, LR-MSCs displayed increased expression of collagen I, fibronectin and α-SMA, the major markers of myofibroblast differentiation, as revealed by western blotting (Fig. 2a,b). To confirm the differentiation, expression of collagen I, α-SMA and fibronectin were further examined by immunofluorescence assay (Fig. 2c).

**Figure 3:**
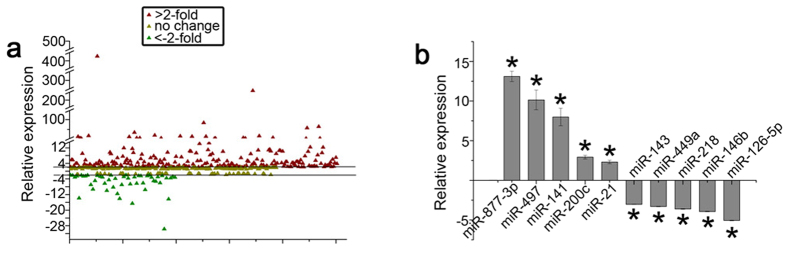
Expression of miRNAs in LR-MSCs is regulated by TGF-β1 (10 ng/ml). (**a**) MiRNA expression in LR-MSCs treated with TGF-β1 for 7 days is shown in scatter plots. Black lines indicate the 2-fold threshold. All values are expressed as fold-changes. (**b**) Altered expression of miRNAs was confirmed by quantitative real-time polymerase chain reaction (Q-PCR). *P < 0.05 vs. Control.

The miRNA expression profiling revealed 299 miRNAs with substantial changes (2^−ΔΔCT^ > 2-fold or <−2-fold) in LR-MSCs following TGF-β1-induced myofibroblast differentiation (see Supplementary Table S1). Of the 299 miRNAs with altered expression, 252 were significantly up-regulated while 47 were evidently down-regulated (P < 0.05) (Fig. 3a). Among the most significantly changed microRNAs were miR-877-3p and miR-126-5p, which were also validated by Q-PCR (Fig. 3b). In this study, miR-877-3p was chosen for further investigation.

### Smad7 is directly regulated by miR-877-3p

**Figure 4:**
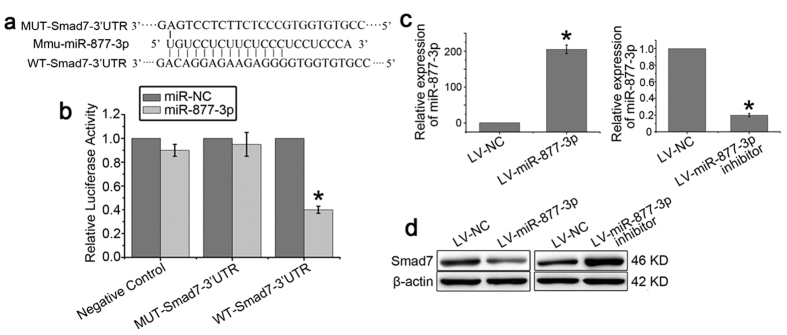
Smad7 is directly regulated by miR-877-3p. (**a**) Smad7 3′UTR fragment containing wild-type (WT) or mutated (MUT) miR-877-3p–binding sequence. (**b**) Luciferase reporter assays were carried out in 293T cells following cotransfection of negative control (NC), WT-Smad7-3′UTR or MUT-Smad7-3′UTR and miR-877-3p or miR-NC as indicated. Firefly luciferase activity was normalized by the Renilla luciferase activity. *P<0.05 vs. miR-NC. (**c**,**d**) LR-MSCs were transiently transfected with LV-miR-877-3p, LV-control, or LV-miR-877-3p inhibitor, followed by incubation for 7 days. The expression of miR-877-3p was measured by Q-PCR (**c**). The expression of Smad7 in LR-MSCs was examined by western blotting (**d**).

The single highly conserved binding site for the miR-877-3p seed sequence in the 3′-UTR of Smad7 is present in mice according to analysis using target prediction programs. To demonstrate a direct interaction between miR-877-3p and Smad7, we cloned 3′UTR sequences that contain the predicted target site (wild type, WT) of miR-877-3p into the pGL3 vector. As a control, we also inserted mutated sequences (mutant type, MUT) into the vector (Fig. 4a). The results showed that co-transfection of miR-877-3p mimics significantly decreased the firefly luciferase activity of the reporter with wild type 3′UTR but not that of the mutant reporter (Fig. 4b), which indicates that miR-877-3p can directly target the 3′UTR of Smad7. To further confirm the relationship between miR-877-3p and Smad7, LR-MSCs were transfected with LV-NC, LV-miR-877-3p-mimic, or LV-miR-877-3p-inhibitor followed by incubation for 7 days. MiR-877-3p was highly increased in cells transfected with LV-miR-877-3p-mimic and decreased in cells transfected with LV-miR-877-3p-inhibitor (Fig. 4c). The expression of Smad7 protein was significantly decreased following transfection with LV-miR-877-3p-mimic and increased with LV-miR-877-3p-inhibitor (Fig. 4d). Taken together, these results indicated that Smad7 was a direct downstream target for miR-877-3p in LR-MSCs.

### miR-877-3p is increased and Smad7 is downregulated in pulmonary fibrosis

**Figure 5:**
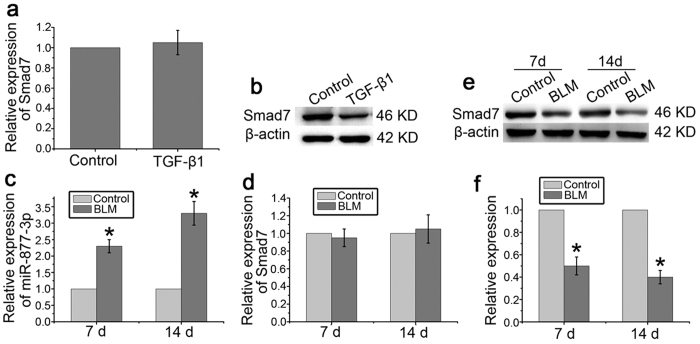
miR-877-3p is increased and Smad7 is downregulated in pulmonary fibrosis. (**a**,**b**) LR-MSCs were cultured with or without TGF-β1 (10 ng/ml) for 7 days. The expression levels of Smad7 during the myofibroblast differentiation of LR-MSCs were measured by Q-PCR (**a**) and western blotting (**b**). (**c**–**f**) Pulmonary fibrosis was induced in bleomycin-treated mice (BLM). Mice (n = 10 in each group) received either saline or bleomycin (5 mg/kg body wt in 50 μl saline) intratracheally. Mice were killed on days 7 and 14. The expression of miR-877-3p and Smad7 was assessed by Q-PCR. *P < 0.05 vs. Control (**c**,**d**). The levels of Smad7 were examined by western blotting (n = 10 per group). Representative gel electrophoresis bands are shown (**e**). The expression levels of proteins were quantified by densitometry and normalized to the expression of β-actin (**f**). Three repeats were performed. *P < 0.05 vs. Control.

As shown in Fig. 5, we found that the expression of miR-877-3p was increased and Smad7 was decreased significantly in bleomycin-treated mice. A similar pattern was also observed during myofibroblast differentiation of LR-MSCs. Although the expression of Smad7 mRNA was not changed significantly, these results indicated that the interaction of miR-877-3p and Smad7 may play an important role in the development of pulmonary fibrosis.

### miR-877-3p can regulate the differentiation of LR-MSCs into myofibroblasts

**Figure 6:**
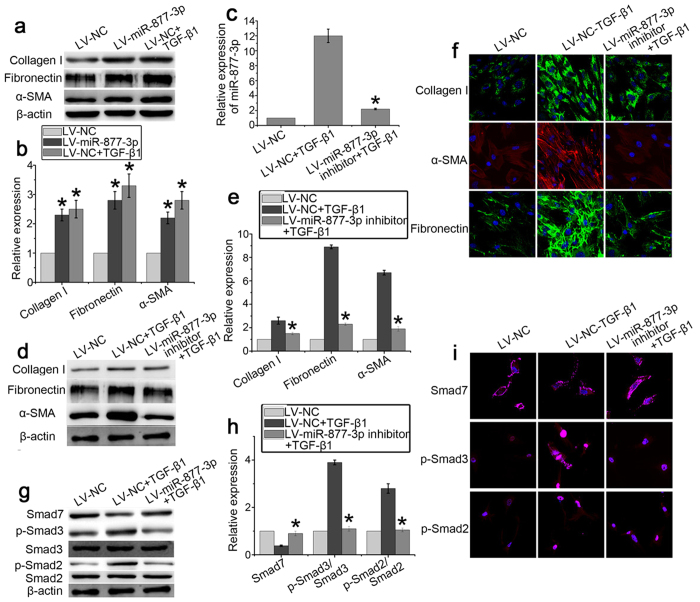
miR-877-3p regulates the differentiation of LR-MSCs into myofibroblasts. LR-MSCs were transiently transfected with LV-miR-877-3p, LV-negative control (NC), or LV-miR-877-3p inhibitor in the presence or absence of TGF-β1 (10 ng/ml) followed by incubation for 7 days. (**a**,**b**) The expression of Collagen I, fibronectin, and α-SMA regulated by LV-miR-877-3p were examined by western blotting. Representative gel electrophoresis bands are shown (**a**). The expression levels of proteins were quantified by densitometry and normalized to the expression of β-actin (**b**). Three repeats were performed. *P < 0.05 vs. LV-NC. (**c**) The expression of miR-877-3p regulated by LV-miR-877-3p inhibitor was measured by Q-PCR. *P < 0.05 vs. LV-NC + TGF-β1. (**d**,**e**) The expression of collagen I, α-SMA, and fibronectin regulated by LV-miR-877-3p inhibitor on LR-MSCs was examined by western blotting. Representative gel electrophoresis bands are shown (**d**). The expression levels of proteins were quantified by densitometry and normalized to the expression of β-actin (**e**). Three repeats were performed. *P < 0.05 vs. LV-NC + TGF-β1. (**f**) The expression of collagen I, α-SMA, and fibronectin regulated by LV-miR-877-3p inhibitor on LR-MSCs was examined by immunofluorescent microscopy (×600). (**g**,**h**) Expression of Smad7, p-Smad2, Smad2, p-Smad3, and Smad3 in LR-MSCs was examined by western blotting. Representative gel electrophoresis bands are shown (**g**). The expression levels of proteins were quantified by densitometry and normalized to the expression of β-actin (**h**). Three repeats were performed. *P < 0.05 vs. LV-NC + TGF-β1. (**i**) Expression of Smad7, p-Smad2, Smad2, p-Smad3, and Smad3 in LR-MSCs was examined by immunofluorescent microscopy (×600).

To determine the effect of miR-877-3p on the differentiation of LR-MSCs, cells were transiently transfected with LV-miR-877-3p, LV-control, or LV-miR-877-3p inhibitor followed by incubation in the presence or absence of TGF-β1 (10 ng/ml) for 7 days. Compared with LV-NC, transfection with LV-miR-877-3p significantly increased the expression of collagen I, fibronectin and α-SMA (Fig. 6a,b), suggesting enhanced myofibroblast differentiation. LV-miR-877-3p-inhibitor can successfully restrain the expression of miR-877-3p (Fig. 6c). After LR-MSCs were transfected with LV-miR-877-3p-inhibitor, TGF-β1-induced expression of collagen I, fibronectin and α-SMA were significantly suppressed (Fig. 6d–f). Next, we determined whether downregulation of miR-877-3p interferes with the TGF-β signaling pathways, particularly with respect to smad protein levels. Although the mRNA expression of Smad7 was unchanged, Smad7 protein was inhibited by TGF-β1. Moreover, after LR-MSCs were transfected with LV-miR-877-3p-inhibitor, the expression of Smad7 was up-regulated. In addition, the phosphorylation of Smad2 and Smad3 in LR-MSCs was markedly enhanced by TGF-β1 but suppressed as a result of the downregulation of miR-877-3p (Fig. 6g–i).

### Smad7 inhibits myofibroblast differentiation of LR-MSCs

**Figure 7:**
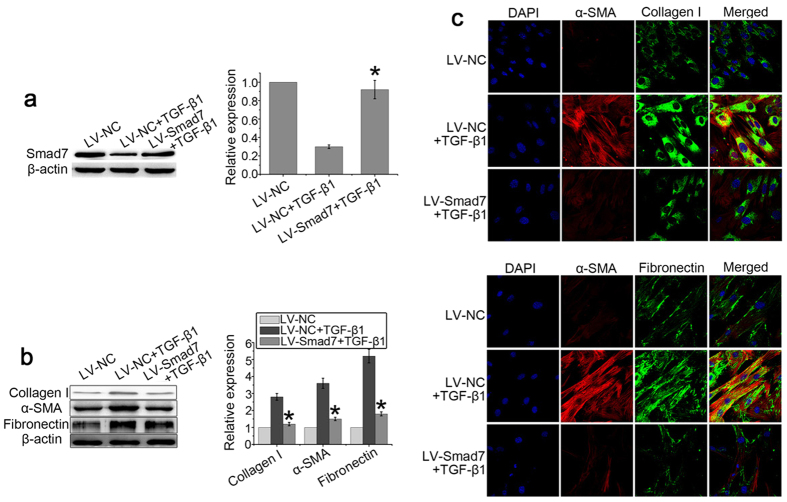
Smad7 inhibits myofibroblast differentiation of LR-MSCs. LR-MSCs were transiently transfected with LV-Smad7 or LV-control in the presence or absence of TGF-β1 (10 ng/ml) followed by incubation for 7 days. (**a**) Expression of Smad7 was measured by western blotting. Representative gel electrophoresis bands are shown (left panel). The expression levels of proteins were quantified by densitometry and normalized to the expression of β-actin (right panel). Three repeats were performed. *P < 0.05 vs. LV-NC + TGF-β1. (**b**) Expression of collagen I, α-SMA, and fibronectin on LR-MSCs was examined by western blotting. Representative gel electrophoresis bands are shown (left panel). The expression levels of proteins were quantified by densitometry and normalized to the expression of β-actin (right panel). Three repeats were performed. *P < 0.05 vs. LV-NC + TGF-β1. (**c**) Expression of collagen I, α-SMA, and fibronectin on LR-MSCs was examined immunofluorescent microscopy (×600).

It has been reported that Smad7 can inhibit epithelial-mesenchymal transition (EMT) and fibroblast-to-myofibroblast differentiation in other cells and pathologies. Therefore, we hypothesized that reduction in Smad7 levels may promote myofibroblast differentiation of LR-MSCs. To investigate the effect of Smad7 on the differentiation of LR-MSCs, we transfected a Smad7 lentivirus vector (LV-Smad7). We found that LV-Smad7 was able to up-regulate Smad7 expression in LR-MSCs (Fig. 7a). Furthermore, transfection of Smad7 significantly decreased TGF-β1-induced expression of α-SMA, fibronectin and collagen I (Fig. 7b,c).

### Downregulation of miR-877-3p inhibits the development of bleomycin-induced pulmonary fibrosis

**Figure 8:**
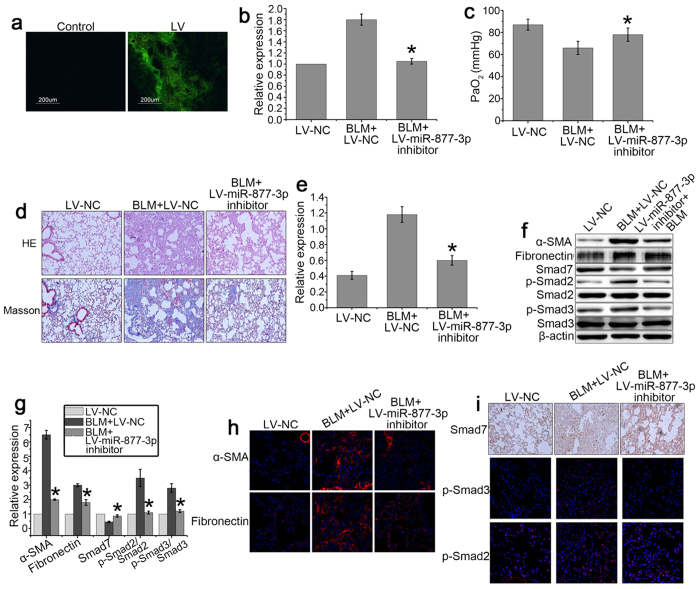
Downregulation of miR-877-3p inhibits the development of bleomycin-induced pulmonary fibrosis. Mice (n = 10 in each group) received either LV-negative control (NC) or LV-miR-877-3p inhibitor (2 × 10^8^ TU in 50 μl saline) intratracheally on three days before intratracheal instillation of bleomycin (BLM) (5 mg/kg body wt in 50 μl saline) or saline. (**a**) mice were killed 14 days after bleomycin instillation. The distribution of lentivirus was detected by fluorescent microscopy. (**b**) Expression of miR-877-3p was measured by Q-PCR. (**c**) PaO2 was assessed by blood gas analysis. Statistical analysis was performed using pairwise t-test. *P < 0.05 vs. BLM + LV-NC. (**d**) Bleomycin-induced pulmonary fibrotic lesions were determined by H&E staining (×100) and Masson’s trichrome stain (×200). (**e**) The effect of LV-miR-877-3p inhibitor on the level of collagen was determined by hydroxyproline measurement. *P < 0.05 vs. BLM + LV-NC. (**f**,**g**) Expression of α-SMA, fibronectin, Smad7, p-Smad2, Smad2, p-Smad3, and Smad3 in the injured lungs was analyzed by western blotting. Representative gel electrophoresis bands are shown (**f**), and the expression levels of the proteins were quantified by densitometry and normalized to the expression of β-actin (**g**). Densitometry data are shown as mean ± SD. *P < 0.05 vs. BLM + LV-NC. (**h**) Expression of α-SMA and fibronectin in the injured lungs was examined by immunofluorescent microscopy (×600). (**i**) Expression of Smad7, p-Smad2, and p-Smad3 in the injured lungs was examined by immunofluorescent microscopy (×600) or immunohistochemistry (×200).

We next explored the inhibitory effects of LV-miR-877-3p-inhibitor on bleomycin-induced pulmonary fibrosis. The GFP of LV-vector can be observed by fluorescent microscopy and we found that LV-miR-877-3p-inhibitor was uniformly distributed in the lung tissue (Fig. 8a). The expression of miR-877-3p was upregulated after bleomycin administration but downregulated significantly following transfection of LV-miR-877-3p-inhibitor (Fig. 8b). The PaO_2_ levels in the bleomycin mice declined significantly and LV-miR-877-3p-inhibitor administration increased PaO2 levels (Fig. 8c). Downregulation of miR-877-3p significantly reduced the severity of pulmonary fibrosis in the lungs compared with negative control group as assessed by H&E and Masson’s trichrome staining (Fig. 8d), as well as the hydroxyproline content assay for collagen deposition (Fig. 8e). Compared with saline controls, expression of fibronectin, α-SMA and collagen I after bleomycin administration was markedly increased, which was significantly inhibited by the transfection of LV-miR-877-3p-inhibitor (Fig. 8f–h). To confirm the inhibitory mechanisms of LV-miR-877-3p-inhibitor in bleomycin-induced pulmonary fibrosis, the expression of Smad7, p-Smad2 and p-Smad3 was measured. The results showed that the pulmonary expression of Smad7 was decreased and the expression of p-Smad2 and p-Smad3 was significantly increased after bleomycin administration. However, regulation of these proteins was abolished in bleomycin-treated mice that received LV-miR-877-3p-inhibitor (Fig. 8f–i).

## Discussion

Various types of cells in the lungs are involved in the fibrotic processes mainly including EMT and fibroblast-to-myofibroblast transdifferentiation^2^. However, more and more studies showed that stem cells are crucial for the development of pulmonary fibrosis^16^. Adult lung tissue contains a population of lung resident MSCs that are precursors of myofibroblasts and also can be induced to differentiate into many other cell types^17^. In our previous study, the LR-MSCs could be induced to differentiate into epithelial cells under specific conditions^18^. However, attention has also been focused on these MSCs with respect to their key role in deleterious remodeling associated with pulmonary fibrosis^19^.

Ectopic expression of microRNAs has been frequently observed in IPF^15^ and studies have shown that let-7, miR-21 and some other miRNAs are closely related to EMT and myofibroblast differentiation. However, the precise roles of microRNAs in the differentiation of LR-MSCs to myofibroblasts and the corresponding molecular mechanisms are still unclear. Therefore, identification of the specific microRNAs and their targets that are essential for cell differentiation may provide valuable therapeutic targets.

TGF-β/Smad signaling has been considered as a key pathway leading to pulmonary fibrosis, which may be an important mechanism that promotes cell differentiation and ECM formation^20^. In our *in vitro* experiment, LR-MSCs were induced to differentiate into a myofibroblast phenotype following treatment with TGF-β1, evidenced by the expression of specific molecular markers of myofibroblasts. The results confirmed the important role of LR-MSCs in the development of pulmonary fibrosis, which has a high expression level of TGF-β1. In the present research, we performed a miRNA microarray and revealed that miR-877-3p was the most significantly upregulated microRNA in the differentiation of LR-MSCs to myofibroblasts. Moreover, miR-877-3p overexpression could also be detected in mouse lungs following administration with bleomycin. The results indicated that miR-877-3p may play a very important role in the development of IPF. Previous studies have reported that miR-877-3p acts as a tumor suppressor^21^, but the role of miR-877-3p in IPF is never reported. In this study, LR-MSCs were transfected with miR-877-3p and we found that overexpression of miR-877-3p could promote the differentiation process. Furthermore, restrain the expression of miR-877-3p could significantly inhibit TGF-β1-induced myofibroblast differentiation. These results support that miR-877-3p expression was significantly correlated with the development of pulmonary fibrosis.

To explore the mechanisms underlying the inhibitory effect of miR-877-3p on myofibroblast differentiation mediated by TGF-β1, we next set out to identify the potential target genes of miR-877-3p. The bioinformatics analysis indicates that Smad7 may be the potential target for miR-877-3p. Moreover, Smad7 is an inhibitory smad in the TGF-β signaling pathway and the suppression of Smad7 could promote the fibrotic process^22^. We demonstrated that miR-877-3p was able to directly target the 3′UTR of Smad7. The discrepancy between the protein expression and the mRNA expression of Smad7 following overexpression of miR-877-3p in LR-MSCs suggests that miR-877-3p may interfere with the transcription of, but not degrade, the Smad7 mRNA.

We further explored the role of miR-877-3p in an *in vivo* study. miR-877-3p inhibitor sequence was inserted into the lentiviral vector and intratracheally injected to the mouse lungs. The transfect effect of lentiviral was confirmed by the observation of GFP expression. After transfection of LV-miR-877-3p-inhibitor, the expression of Smad7 was increased in the bleomycin-induced mouse pulmonary fibrosis model, and the development of pulmonary fibrosis was profoundly restrained. Furthermore, we demonstrated that the expression of p-Smad2 and p-Smad3, which are the downstream proteins of Smad7 signaling were decreased. Our data demonstrated that miR-877-3p may promote pulmonary fibrosis by down-regulating the expression of Smad7, which is reported to serve as an inhibitor in pulmonary fibrosis^23^.

Increasing evidence has shown that Smad7 is correlated to EMT and fibrosis^24^,^25^. Some researchers have reported that Smad7 could stabilize β-catenin, which is an essential mediator of the canonical Wnt signaling pathway, by binding at the plasma membrane rather than being transported into the nucleus to activate Wnt signaling^26^. In our previous study, we have shown that inhibition of Wnt/β-catenin signaling could repair bleomycin-induced lung fibrosis^27^. To investigate the role of Smad7 in the myofibroblast differentiation of LR-MSCs, we up-regulated the Smad7 gene expression and found that Smad7 could inhibit the differentiation process. This indicated that up-regulation of Smad7 may contribute to the inhibition of lung fibrosis.

In conclusion, we explored the role of miRNA in the myofibroblast differentiation of LR-MSCs and the development of bleomycin-induced lung fibrosis. We found that miR-877-3p is highly up-regulated in the myofibroblast differentiation of LR-MSCs and in the fibrotic lung. More importantly, we found that miR-877-3p sequestration inhibited the myofibroblast differentiation of LR-MSCs and attenuates bleomycin-induced lung fibrosis by targeting Smad7. Our data implicate a potential application of miR-877-3p as a fibrosis suppressor in pulmonary fibrosis therapy and also as a fibrosis marker for predicting prognosis.

## Methods

### Ethics statement

The animal experiments were performed according to the Guide for the Care and Use of Laboratory Animals (The Ministry of Science and Technology of China, 2006) and all experimental protocols were approved under the animal protocol number SYXK (Su) 2009–0017 by the Animal Care and Use Committee of Nanjing University.

### Isolation of LR-MSCs

Lung single-cell suspensions were prepared from the lungs of at least 5 C57BL/6 mice (4–6 weeks old). In brief, the lung parenchyma from mice was digested by fine mincing with a razor blade, followed by incubation in an enzyme mixture containing 0.2% collagenase I (Sigma, USA), 2.4 U/ml dispase (Sigma, USA) and 0.001% DNAse (Sigma) for 1 h at 37 °C with shaking. This suspension was filtered through 100-μm and 40-μm filters, centrifuged, and depleted of red blood cells by RBC lysis buffer. Cells were resuspended in PBS at 1 × 10^7^ cells/ml and stained for Sca-1, CD45 and CD31 followed by sorting using the AutoMACS cell separator system (Miltenyi Biotec, Bergisch Gladbach, Germany).

Freshly isolated LR-MSCs were cultured at a concentration higher than 10^5^ cells/ml with DMEM containing 10% fetal bovine serum, 4% L-glutamine, 1% nonessential amino acids, and 1% penicillin and streptomycin, and maintained in a humidified atmosphere of 95% air, 5% CO_2_ at 37 °C. The culture medium was changed every 48 h, and cells were passaged 1:2 using 0.25% trypsin when they reached 70–90% confluence.

### Flow cytometric analysis

To determine the expression of various surface markers, LR-MSCs following the first passage were incubated with fluorescent antibodies at 37 °C for 40 min in the dark followed by two washes with PBS. Flow cytometry was performed on a FACS CaliburTM flow cytometer and the data were analyzed using the Paint-A-Gate software (Becton Dickinson). The antibodies used were: FITC-conjugated anti-Ly-6A/E (Sca-1), PE-conjugated anti-CD45 and PE-conjugated anti-CD34 (eBioscience, San Diego, CA); APC-conjugated anti-CD31 and PE-conjugated anti-CD29 (Bio-legend, San Diego, CA).

### Myofibroblast differentiation of LR-MSCs

To induce differentiation of LR-MSCs into myofibroblasts, LR-MSCs were incubated with 10 ng/ml TGF-β1. On days 3, 7 and 14, LR-MSCs were harvested for analysis of the myofibroblast markers.

### RNA purification

Cells or lung tissues were harvested for RNA purification. Total RNA was isolated using the mirVana miRNA Isolation Kit (Invitrogen, Carlsbad, CA) for miRNA analysis or using the TRIzol reagent (Invitrogen) for mRNA evaluation.

### Low-Density miRNA taqman array and bioinformatics

Cells incubated in the absence or presence of 10 ng/ml TGF-β for 7 days were harvested and total RNA was isolated immediately from 10^6^ cells. The miRNA microarray analysis was performed by low-density miRNA Taqman array (Invitrogen) according to the manufacturer’s instructions. Data was analyzed as previously described^28^. miRNAs with expression values of less than 0.5 (log scale)-fold-change compared to controls were considered as under-expressed while those with a value of greater than 2 were regarded as overexpressed. Experiments that included three technical replicates were conducted twice and in total six data sets per condition were obtained. Potential targets for miRNA action were predicted by using putative targets generated from miRWalk 2.0 (http://www.umm.uni-heidelberg.de/apps/zmf/mirwalk/).

### Quantitative real-time polymerase chain reaction (Q-PCR)

One microgram of total RNA was used for cDNA synthesis. Reverse transcription was performed using the Superscript III first-strand synthesis system (Invitrogen) or the Taqman microRNA RT kit (Invitrogen) on a Veriti 96-Well Fast Thermal Cycler (Applied Biosystems, Grand Island, NY). Q-PCR was performed using the SYBR GreenER Q-PCR kit (Invitrogen) or the Taqman microRNA assay and universal PCR Mastermix (Invitrogen) using a 7900 HT Fast Real-Time PCR System (Applied Biosystems). Total RNA was isolated from cells in three independent culture plates and analyzed in duplicate using Q-PCR. All the procedures were repeated for three times. The relative quantification values for each miRNA and mRNA were calculated by the 2^−ΔΔCt^ method using U6 and GAPDH as an internal reference, respectively. Primer pairs of mRNA used are as following: Smad7 forward 5′-GGGCTTTCAGATTCCCAACTT-3′ and reverse 5′-AGGGCTCTTGGACACAGTAGA-3′; GAPDH forward 5′-AGGTCGGTGTGAACGGATTTG-3′ and reverse 5′-TGTAGACCATGTAGTTGAGGTCA-3′.

### Vector construction and transfection

DNA fragment for miR-877-3p or Smad7 was amplified from genomic DNA and inserted into the Age I/EcoR I site of the lentiviral expression vector pGCSIL-GFP (GeneChem, Shanghai, China). Nucleotide sequences of murine miR-877-3p inhibitor used is as following: CCGGTGGGAGGAGGGAGAAGAGGACATTTTT. The Smad7 expression vector was constructed by inserting the ORF sequence into the pGCL vector (GeneChem, Shanghai, China). Transfection was performed according to the manufacture’s protocol with the viruses at a multiplicity of infection of 100 in the presence of 5 μg/ml polybrene (Sigma, MO). In the present study, the infection efficiency of lentivirus was over 90%. No significant cell death was observed after virus infection.

### Luciferase reporter assay

Luciferase activity assay was performed using the Dual-Luciferase Reporter Assay System (Promega, Madison, WI) according to the manufacturer’s instructions. 293T cells of 85–90% confluence were seeded in 96-well plates. For Smad7 (NM_001042660) 3′UTR luciferase reporter assay, 100 ng wild type or mutant luciferase reporter constructs (termed WT or MUT) were co-transfected into 293T cells in a 96-well plate with 50 ng pGL3-Promoter-UTR and 10 ng pRLTK using lipofectamine 3000 (Invitrogen). Luciferase activity assay was performed 48 hours after transfection using the Dual-Luciferase Assay System. Firefly luciferase activity was normalized to the corresponding Renilla luciferase activity. All the experiments were performed three times.

### Immunofluorescent staining

Immunofluorescence analysis of LR-MSCs or lung tissues was performed as described previously^29^. The following primary antibodies were employed: Rabbit anti-collagen I, mouse anti-α-smooth muscle actin (α-SMA), rabbit anti-fibronectin, rabbit anti-Smad7, rabbit anti-p-Smad2, rabbit anti-p-Smad3 (all antibodies were purchased from Abcam, Cambridge, MA). Alexa Fluor 488 or 594-conjugated goat anti-rabbit antibody (Invitrogen) was used as a secondary antibody. The nucleus are staining with 4′, 6-diamidino-2-phenylindole (DAPI) (Sigma, MO). The images were captured using a confocal fluorescence microscope (Olympus, Tokyo, Japan).

### Western blotting

Proteins were purified from either LR-MSCs or lung tissues. Western blotting analysis of cellular lysates was performed as previously described^30^. Proteins were separated using 12% SDS-polyacrylamide gel electrophoresis and were electrophoretically transferred to polyvinylidene fluoride (PVDF) membranes using standard procedures. The following primary antibodies were employed: Rabbit anti-collagen I, rabbit anti-α-SMA, rabbit anti-fibronectin, rabbit anti-Smad7, rabbit anti-p-Smad2, rabbit anti-p-Smad3, rabbit anti-Smad2, rabbit anti-Smad3, rabbit anti- FSP-1, and mouse anti-β-actin (Abcam). Horseradish peroxidase-conjugated goat anti-rabbit/mouse IgG (Boster, Wuhan, China) was used as a secondary antibody. Immunoreactive protein bands were detected using an Odyssey Scanning System (LI-COR). Representative gel electrophoresis bands are shown in figures, and the expression levels of the proteins were quantified by densitometry using Image J and normalized to the expression of β-actin.

### Bleomycin-induced mouse pulmonary fibrosis model

Male C57BL/6 mice (6–7 weeks old) were purchased from the Medical School of Yangzhou University (Yangzhou, China). All mice were maintained under standard conditions with free access to water and laboratory rodent food. For the evaluation of the effect of miR-877-3p on bleomycin-induced pulmonary fibrosis, mice were anesthetized with pentobarbital sodium (3 mg/kg). Next, mice received a single, slow intratracheal injection of 2 × 10^8^ TU LV-negative control (NC) or LV-miR-877-3p with MicroSprayer (Penn-Century, Wyndmoor, PA). The GFP was insert in the LV-vector. Three days later, mice received 5 mg/kg bleomycin (Nippon Kayaku Co., Tokyo, Japan) dissolved in 50 μl of saline on day 0. Control mice received 50 μl of saline instead. Mice were sacrificed on days 14. Mouse lungs were obtained for the analysis of histopathology, collagen content, Q-PCR and western blotting.

### Pulmonary function measurements

Mice were analyzed for arterial blood gas contents before being sacrificed. All procedures were employed in compliance with the Guide for the Care and Use of Experimental Animals formulated by the National Society for Medical Research. The i-STAT Portable Clinical Analyzer and the i-STAT G7+ cartridges were used (Abbott Point of Care, Chicago, IL). Arterial blood was sampled from the left ventricle of the mice (n = 6 in each group), and then it was introduced into the sample well and allowed to fill by passive movement to the indicated level (80–100 μl volume). The cap on the sample well was closed and the cartridge was inserted into the analyzer. After successful completion of the calibration and analysis cycles, the PaO_2_ values were recorded.

### Histology

Lower left lungs were fixed in 4% (w/v) neutral phosphate-buffered paraformaldehyde for 24 h, dehydrated, transparentized and embedded in paraffin. Lung tissues were cut into 5-μm sections which were stained with hematoxylin-eosin (H&E) for structured observation, or with Masson’s trichrome stain for detection of collagen deposits. Determination of hydroxyproline content was carried out using a kit from Nanjing JianCheng Bioengineering Institute (Nanjing, China) according to the instructions by the manufacturer.

### Immunohistochemistry analysis

The slides were gotten rid of paraffin, subjected to antigen retrieval, and quenching of endogenous peroxidase activity using 3% (v/v) H_2_O_2_ for 10 min. Immune complexes were visualized using suitable peroxidase-coupled secondary antibodies, according to the manufacturer’s protocol (PV-9000 2-step plus poly-HRP anti-mouse/rabbit IgG detection system; GBI, USA). The rabbit anti- Smad7 primary antibody (Abcam, Cambridge, MA) was employed. The secondary antibody incubated were horseradish peroxidase-conjugated goat anti-rabbit IgG (Boster, Wuhan, China).

### Statistical analysis

Data were expressed as mean ± standard deviation from at least three independent experiments. The differences between groups were analyzed for significance (*P* < 0.05) by t test when only two groups were compared or by one-way analysis of variance (ANOVA) or when more than two groups were compared. All statistical analysis was performed using SPSS for windows version 11.0 (SPSS, Chicago, IL).

## Additional Information

**How to cite this article**: Wang, C. *et al*. miR-877-3p targets Smad7 and is associated with myofibroblast differentiation and bleomycin-induced lung fibrosis. *Sci. Rep.* **6**, 30122; doi: 10.1038/srep30122 (2016).

## Supplementary Material

Supplementary Information
