# Supplementary material for: miR-877-3p targets Smad7 and is associated with myofibroblast differentiation and bleomycin-induced lung fibrosis
Source: Sci Rep. 2016 Jul 22;6:30122. doi: 10.1038/srep30122 (PMC4957095; doi:10.1038/srep30122)
Supplement: Supplementary Information [file srep30122-s1.pdf]

Title: miR-877-3p targets Smad7 and is associated with myofibroblast differentiation and bleomycin-induced lung fibrosis

Authors: Cong Wang[1, 2], Shen Gu[1, 2], Honghui Cao[1, 2], Zutong Li[1, 2], Zou Xiang[3], Kebin Hu[4], Xiaodong Han[1, 2*]

Affiliation of each author:

[1] Immunology and Reproduction Biology Laboratory & State Key Laboratory of Analytical Chemistry for Life Science, Medical School, Nanjing University, Nanjing, Jiangsu 210093, China

[2] Jiangsu Key Laboratory of Molecular Medicine, Nanjing University, Nanjing, Jiangsu 210093, China

[3] Department of Microbiology and Immunology, Mucosal Immunobiology and Vaccine Research Center, Institute of Biomedicine, University of Gothenburg, Gothenburg, Sweden

[4] Department of Medicine, Division of Nephrology, Penn State University College of Medicine, Hershey, Pennsylvania 17033

Supplemental Information

Supplementary Table S1 The entire list of profiled miRNA in lung resident mesenchymal stem cells following TGF-β1-induced myofibroblast differentiation

Assay	Fold-change(TGF-β1/Control)	P-value
mmu-miR-877-3p	423.180	0.0218507
mmu-miR-497	289.158	0.0218864
mmu-miR-141	100.550	0.0219615
mmu-miR-1188	95.116	0.0220154
mmu-miR-135a	87.659	0.0221247
mmu-miR-541	75.057	0.0222315
mmu-miR-466J	69.987	0.0223636
rno-miR-327	68.916	0.0224444
mmu-miR-1956	65.876	0.0224753
mmu-miR-369-5p	59.520	0.0225365
mmu-miR-342-5p	52.496	0.0225556
mmu-miR-322#	51.806	0.0226004
rno-miR-99a#	46.179	0.0228223
mmu-miR-551b	42.938	0.0228599
mmu-miR-190	41.566	0.0228745
mmu-miR-202-3p	36.158	0.0228974
mmu-miR-493	35.821	0.0231079
mmu-miR-196b	34.326	0.0231122
mmu-miR-448	30.534	0.0231566
rno-miR-758	30.285	0.0233726
mmu-miR-295	27.431	0.0233974
mmu-miR-1186	27.345	0.0234503
mmu-miR-200c	24.499	0.0234544
mmu-miR-297a#	21.878	0.0234611
mmu-miR-136#	20.736	0.0235529
rno-miR-743b	20.307	0.0237209
mmu-miR-672	17.205	0.023817
mmu-miR-1982.2	16.930	0.0238287
mmu-miR-1939	16.397	0.023875
mmu-miR-669n	16.286	0.0238942
mmu-miR-16#	16.274	0.0239037
mmu-miR-1894-3p	15.404	0.0240543
mmu-miR-301b	14.420	0.0241593
hsa-miR-33a#	14.152	0.024188
mmu-miR-129-5p	12.345	0.024388
mmu-miR-107	12.262	0.0244387

mmu-miR-665	11.897	0.0244408
mmu-miR-542-5p	11.583	0.0244711
mmu-miR-1933-5p	11.077	0.0244787
hsa-miR-30d#	10.949	0.0245249
mmu-miR-193	10.863	0.0245368
mmu-miR-1960	10.809	0.0247115
mmu-miR-188-5p	10.632	0.0248069
mmu-miR-185	10.552	0.0248759
mmu-miR-345	10.359	0.0249034
mmu-miR-1928	10.294	0.0249208
mmu-miR-183	10.141	0.0249339
hsa-miR-23a#	10.100	0.0249456
mmu-miR-344	10.097	0.0249628
mmu-miR-133a	10.078	0.0249926
mmu-miR-412	9.701	0.0251105
mmu-miR-708	9.619	0.0252215
mmu-miR-542-3p	9.449	0.0252373
mmu-miR-10a	9.406	0.0253257
mmu-miR-339-5p	9.279	0.0254089
rno-miR-743a	9.020	0.0256511
mmu-miR-1897-3p	8.975	0.0256968
hsa-miR-29a#	8.951	0.025715
mmu-miR-706	8.888	0.0257295
mmu-miR-1982.1	8.870	0.0257795
mmu-miR-489	8.616	0.0259303
rno-miR-29c#	8.329	0.02599
mmu-miR-1193	8.291	0.0260915
mmu-miR-466k	7.814	0.0261578
mmu-miR-138#	7.765	0.0261794
mmu-miR-669a	7.625	0.0261939
mmu-miR-376a#	7.552	0.0262237
mmu-miR-367	7.251	0.0264607
hsa-miR-28-3p	7.003	0.0265669
mmu-miR-208	6.819	0.0266524
mmu-miR-467b	6.813	0.0266544
mmu-miR-673-3p	6.804	0.02688
mmu-miR-466g	6.606	0.0270587
mmu-miR-491	6.355	0.0271583
mmu-miR-592	6.264	0.0271869
mmu-miR-1961	6.105	0.0272028
mmu-let-7a	6.094	0.0272067
mmu-miR-423-5p	6.063	0.0273226
mmu-miR-21	5.919	0.027367

mmu-miR-299	5.832	0.0274791
hsa-miR-27b#	5.785	0.0275249
U87	5.731	0.0276101
mmu-miR-299	5.703	0.0276448
rno-miR-382#	5.684	0.0277111
hsa-miR-455	5.521	0.0278347
mmu-miR-652	5.487	0.0278601
mmu-miR-125b#	5.476	0.0278804
mmu-miR-194	5.470	0.0279679
mmu-miR-331-5p	5.453	0.0282164
mmu-miR-199b	5.355	0.0283966
mmu-miR-337-5p	5.312	0.02844
mmu-miR-547	5.236	0.0284422
mmu-miR-1905	5.219	0.0285794
mmu-miR-873	5.196	0.0285853
mmu-miR-2183	5.167	0.0286859
mmu-miR-292-3p	5.057	0.0287
mmu-miR-199a-5p	4.985	0.0287258
hsa-miR-149	4.978	0.0287598
mmu-miR-346	4.930	0.0287915
mmu-miR-150	4.846	0.0287973
mmu-miR-700	4.841	0.029056
mmu-miR-181a	4.806	0.0290699
hsa-miR-213	4.775	0.0291006
mmu-miR-485-3p	4.738	0.029156
mmu-miR-687	4.729	0.0291958
mmu-let-7c-1#	4.613	0.0292239
mmu-miR-376b#	4.571	0.029264
mmu-miR-463	4.471	0.0292921
mmu-miR-805	4.429	0.029507
mmu-miR-496	4.392	0.029511
hsa-miR-196a	4.341	0.0295309
mmu-miR-15a#	4.284	0.029557
mmu-miR-434-5p	4.281	0.0297581
hsa-miR-493-3p	4.279	0.0297729
mmu-miR-155	4.267	0.0297864
mmu-miR-146a	4.257	0.0298448
mmu-miR-1839-5p	4.234	0.0298496
rno-miR-664	4.164	0.0298942
mmu-miR-31#	4.133	0.0299634
mmu-miR-1896	4.088	0.0300076
snoRNA135	4.000	0.0300426
mmu-miR-215	3.999	0.0300743

hsa-miR-140-3p	3.961	0.0300919
hsa-miR-590-3P	3.957	0.0301587
snoRNA135	3.896	0.0301653
mmu-miR-878-3p	3.887	0.0302741
mmu-miR-10b	3.880	0.0303482
hsa-miR-136#	3.840	0.0304846
mmu-miR-674#	3.832	0.0306169
mmu-miR-324-5p	3.797	0.0306496
rno-miR-532-5p	3.784	0.0306656
mmu-miR-196a#	3.781	0.0306882
hsa-miR-875-5p	3.668	0.0308036
mmu-miR-2146	3.652	0.0308245
mmu-miR-743a	3.635	0.0308853
mmu-miR-192	3.632	0.0309244
hsa-miR-154#	3.607	0.0309309
mmu-let-7c	3.561	0.0311791
rno-miR-20b	3.550	0.031236
mmu-miR-340-5p	3.537	0.0312462
mmu-miR-182	3.531	0.0312813
hsa-miR-411#	3.520	0.0312951
mmu-miR-696	3.499	0.0312992
hsa-miR-22#	3.456	0.0314078
mmu-miR-18a	3.437	0.0314171
hsa-miR-421	3.410	0.0314175
mmu-miR-467d	3.407	0.0314423
mmu-miR-467a	3.353	0.0314651
mmu-miR-543	3.343	0.0314674
mmu-miR-467a	3.341	0.0314935
mmu-miR-1948	3.281	0.0315254
mmu-miR-101b	3.271	0.0315549
mmu-miR-1971	3.196	0.0316617
mmu-miR-2182	3.196	0.0317061
mmu-miR-669m	3.187	0.0317287
mmu-miR-1944	3.115	0.0317701
mmu-miR-29b	3.064	0.0317992
hsa-miR-151-5P	3.048	0.0319828
mmu-miR-881#	3.037	0.0319991
mmu-miR-370	3.012	0.0320717
rno-miR-148b-5p	3.002	0.0320789
mmu-let-7d	2.990	0.0321423
mmu-let-7e	2.979	0.0321748
hsa-miR-200c	2.942	0.0322222
rno-miR-350	2.935	0.0323288

rno-miR-381	2.926	0.0323519
hsa-miR-27a#	2.911	0.0323519
mmu-miR-2134	2.906	0.0323544
mmu-miR-130b#	2.886	0.0323551
mmu-miR-467b	2.856	0.0323618
mmu-miR-487b	2.850	0.0323768
mmu-miR-1937b	2.832	0.0324939
mmu-miR-361	2.827	0.0325479
mmu-miR-465a-5p	2.794	0.0325479
mmu-miR-125b-5p	2.751	0.0325479
mmu-miR-214	2.747	0.0325479
mmu-miR-433	2.721	0.0325479
mmu-miR-804	2.711	0.0325767
snoRNA202	2.693	0.03258
mmu-miR-101a	2.647	0.0327789
mmu-miR-466a-3p	2.644	0.0328365
rno-miR-343	2.612	0.0328755
mmu-miR-290-5p	2.600	0.0329161
mmu-miR-7a	2.588	0.0329433
mmu-miR-495	2.579	0.0329484
mmu-miR-467F	2.551	0.0329755
mmu-miR-2135	2.539	0.033041
mmu-let-7a#	2.496	0.0331154
mmu-miR-224	2.494	0.0331691
mmu-miR-345-5p	2.493	0.0333205
mmu-miR-128a	2.489	0.0336463
mmu-miR-125b-3p	2.477	0.0336492
hsa-miR-183#	2.440	0.0337056
mmu-miR-34c#	2.426	0.0337302
hsa-miR-744#	2.422	0.0337489
mmu-miR-669l	2.418	0.033793
hsa-miR-214	2.415	0.0338634
mmu-miR-127	2.412	0.033881
hsa-miR-200c#	2.411	0.0339174
mmu-miR-328	2.406	0.0339265
mmu-miR-503	2.404	0.0340003
mmu-miR-409-5p	2.403	0.0340498
mmu-miR-744	2.402	0.0340507
mmu-miR-17#	2.377	0.0341888
mmu-miR-1274a	2.368	0.0342138
mmu-miR-98	2.363	0.0342577
mmu-miR-1949	2.362	0.0342752
rno-miR-7a#	2.358	0.0342903

mmu-miR-470#	2.344	0.0343022
mmu-miR-216a	2.343	0.0343145
mmu-miR-23b	2.340	0.0343164
hsa-miR-15b#	2.331	0.0343541
mmu-miR-148b	2.319	0.034433
mmu-miR-329	2.303	0.0344639
rno-miR-7#	2.293	0.0346168
mmu-miR-376a	2.282	0.0346824
mmu-miR-34c	2.278	0.0346858
rno-miR-632	2.272	0.0347518
mmu-miR-193#	2.246	0.0347772
hsa-miR-106b#	2.243	0.0348278
mmu-miR-382	2.227	0.0348347
mmu-miR-20a	2.215	0.034852
hsa-miR-22	2.210	0.0348879
mmu-miR-872#	2.208	0.0350129
mmu-miR-409-3p	2.205	0.0350149
mmu-miR-15a	2.201	0.0350585
mmu-miR-24-2#	2.196	0.0351828
mmu-let-7b	2.184	0.0353449
mmu-miR-211	2.167	0.035377
hsa-miR-93#	2.165	0.0358401
mmu-miR-340-3p	2.159	0.0358841
mmu-miR-210	2.152	0.035973
mmu-miR-140	2.150	0.0362217
mmu-miR-362-3p	2.142	0.0362502
mmu-miR-1937c	2.141	0.0363422
mmu-miR-218-1#	2.138	0.0363626
mmu-miR-139-5p	2.120	0.0363762
hsa-miR-378	2.118	0.0365431
hsa-miR-340	2.101	0.0365655
mmu-miR-667	2.096	0.0367463
mmu-miR-337	2.096	0.0367481
mmu-miR-881	2.075	0.0370294
hsa-miR-423-3P	2.052	0.0370464
mmu-miR-374-5p	2.039	0.037054
snoRNA202	2.035	0.0371403
mmu-miR-1969	2.034	0.0371968
mmu-miR-712	2.030	0.0373512
mmu-miR-467H	2.028	0.0374167
mmu-miR-380-5p	2.012	0.0374295
mmu-miR-674	2.003	0.0375104
mmu-miR-204	2.001	0.037581

mmu-miR-685	2.000	0.0375979
mmu-miR-1903	0.489	0.0163889
rno-miR-146B	0.479	0.0164098
mmu-miR-9	0.478	0.0166278
mmu-miR-503#	0.476	0.0166351
mmu-miR-697	0.471	0.0166767
rno-miR-673	0.464	0.0166852
mmu-miR-721	0.442	0.0167017
mmu-miR-222	0.434	0.0167518
mmu-miR-1306	0.430	0.0167613
mmu-miR-218	0.414	0.0167781
mmu-miR-1981	0.409	0.0169408
hsa-miR-9#	0.400	0.0171027
rno-miR-345-3p	0.399	0.017162
rno-miR-125b#	0.390	0.0172665
mmu-miR-103	0.367	0.017272
mmu-miR-676#	0.365	0.0174461
mmu-miR-449a	0.353	0.017475
hsa-miR-143	0.335	0.0175381
mmu-miR-339-3p	0.328	0.0177589
mmu-miR-669D	0.321	0.0177835
rno-miR-351	0.319	0.0178467
mmu-let-7g#	0.316	0.0179363
mmu-miR-187	0.315	0.0179804
mmu-miR-1198	0.280	0.0180724
hsa-miR-10a#	0.280	0.0182429
hsa-miR-425	0.276	0.0183714
mmu-miR-466h	0.262	0.0184475
mmu-miR-590-5p	0.222	0.0185203
mmu-miR-741	0.205	0.018544
mmu-miR-181c	0.201	0.0186175
mmu-miR-154	0.190	0.0186945
mmu-miR-137	0.166	0.0188221
mmu-miR-200a	0.164	0.0188733
mmu-miR-668	0.159	0.0189153
mmu-miR-302a	0.158	0.0189202
mmu-miR-375	0.151	0.0190783
mmu-miR-770-3p	0.144	0.0191027
mmu-miR-449b	0.144	0.0191636
mmu-miR-467e#	0.121	0.0192722
mmu-miR-742#	0.108	0.0192877
mmu-miR-666-3p	0.101	0.0194622
mmu-miR-450a-5p	0.086	0.0196347

rno-miR-339-3p	0.072	0.0199237
mmu-miR-450a-3p	0.071	0.0201402
hsa-miR-1197	0.070	0.0201507
mmu-miR-362-5p	0.060	0.0203493
mmu-miR-126-5p	0.034	0.0204822
